# Changes in public–police cooperation following the murder of George Floyd

**DOI:** 10.1093/pnasnexus/pgac189

**Published:** 2022-09-10

**Authors:** P Jeffrey Brantingham, George Mohler, John MacDonald

**Affiliations:** Department of Anthropology, University of California, Los Angeles, 341 Haines Hall, Los Angeles, CA 90230, USA; Computer Science Department, Boston College, 245 Beacon Street, Chestnut Hill, MA 02467-3859, USA; Department of Criminology, University of Pennsylvania, 558 McNeil Building, Philadelphia, PA 19104-6286, USA

**Keywords:** policing, race, crime, calls-for-service, regression discontinuity design

## Abstract

The murder of George Floyd triggered a broad social response and noticeable shift in public opinion of policing. Since policing is dependent upon public cooperation, a question is whether the shift in opinion had an effect on police calls-for-service. Data from Los Angeles and New York City are evaluated using a regression discontinuity design. Violent crime, property crime, and quality-of-life disorder calls are analyzed to address differences in reporting norms. The role of racial–ethnic composition of local areas is assessed by examining census tracts where the majority of the residential population is Asian, Black, Hispanic, or White. Following the murder of George Floyd, in New York City violent crime, property crime, and quality-of-life calls all increased significantly. In Los Angeles, quality-of-life calls for service decreased significantly. The increase in violent crime calls observed in New York was greatest in areas where the majority of residents are Black, whereas the increase in property crime calls was the greatest in areas where a majority of residents are White. The decrease in quality-of-life calls observed in Los Angeles was in areas where the majority of residents are White. In both cases, the effect was relatively short-lived.

Significance Statement Mistrust of police should matter for people's willingness to report crime, yet many studies find that mistrust is overridden by other concerns. If any event was likely to have pushed trust below a threshold that fundamentally reduced public–police cooperation to report crimes, the murder of George Floyd should qualify. The murder did have an immediate impact on calls to the police in both Los Angeles and New York, the two largest cities in the United States, but the effect was relatively short-lived and, in New York City, in the opposite direction of a prediction that a decline in trust in the police would reduce people's willingness to call the police to report crimes and quality-of-life offenses.

## Introduction

Calling the police is a basic form of public cooperation with the justice system (1). However, cooperation with the police is not necessarily indicative of trust in the police, or perceived police legitimacy (2, 3). Indeed, people may call the police because they have no credible alternative to resolving an immediate problem they face. A critical question is how far must trust in the police fall before people choose not to call, even in the absence of a credible alternative. The answer to this question is central to understanding how community-led solutions to crime and disorder might fill the gap if police response to calls-for-service were to be scaled back from the present form.

The murder of George Floyd on 2020 May 25 by a Minneapolis police officer is but one example in recent years of a police incident of deadly force that spurred widespread media coverage and public protests. The killings of Breonna Taylor in Louisville, KY, Jamar Clark in Minneapolis, MN, Freddie Gray in Baltimore, MD, Tamir Rice in Cleveland, OH, Michael Brown in Ferguson, MO, and Eric Garner in New York, NY, among others, brought people into the streets with calls for accountability, police reform, and, occasionally, abolition of police (4). The murder of George Floyd, captured in graphic detail on video, broadened public protests and amplified calls to “defund the police” to a previously unprecedented degree (5, 6). It is reasonable to suggest that the murder of George Floyd marked a nadir in public opinion of the police. More than any moment in recent history, we might expect that the murder of George Floyd finally pushed trust low enough to have a major impact on public cooperation with the police and the public's willingness to report crimes and quality-of-life disorder offenses.

Here, we examine the above proposition using police calls-for-service data from 2019 to 2021 in Los Angeles, CA, and New York, NY, to determine whether the murder of George Floyd and the subsequent social response had an impact on day-to-day cooperation between the public and the police. Any such changes in cooperation with the police are likely to vary based upon neighborhood and demographic characteristics (2, 7). The prevailing assumption is that cooperation should fall more precipitously in communities that already perceive that they suffer from heavy-handed policing. We therefore examine how the impact of the murder of George Floyd varied by communities where a majority of residents are Asian, Black, Hispanic, or White in Los Angeles and New York. We also examine the impact in more diverse communities where no one racial–ethnic group comprises a majority.

## Background

The modern policing model is dependent upon cooperation with the public. Without public reporting of crimes, most crime would go undetected by the police. While it seems obvious that trust in police enhances the willingness of people to report crime, it has been noted that the absence of trust does not necessarily mean the absence of reporting (2, 3, 8). If people lack a viable alternative to resolving a crime themselves, then they may still turn to the police for help, in spite of low trust in policing. This latter hypothesis has been offered as one explanation for the well-documented empirical pattern that the highest crime reporting rates generally arise in the communities that should trust the police the least. Specifically, Black victims of crime tend to report victimization at higher rates than Hispanic/Latino victims, and Hispanic/Latino victims at higher rates than White victims (2, 8–11).

Numerous other factors may override issues of trust or legitimacy in the decision to call the police. Variation between neighborhoods in crime rates and local social norms appear to matter. Huebner et al. (12), for example, document in detail how the tendency to call the police about “shots fired” tends to be proportionally lower in the places with the most gunfire, as determined by acoustic gunshot detectors. People in high-crime neighborhoods may be desensitized to the sound of gunfire and its consequences. The tendency to report crime is thus seen as a learned response tied to local contexts. Learned social norms may also matter. Jackson et al. (7), for example, found that individuals are more (less) willing to cooperate with the police based on whether they have internalized neighborhood norms that they *should* (should not) help the police fight crime and disorder. The legal cynicism that underlies an unwillingness to call the police may create conditions that allow crime to flourish (13).

Willingness to call the police and to seek help in general also appears to vary by both crime type and the specific characteristics of victims and offenders (8, 14, 15). The probability that people call the police is generally lowest for sex-based offenses (15), though the situation has improved dramatically over the years (8). Burglary and aggravated assault generate calls to the police with about the same probability. Robbery and car theft tend to generate calls to the police with the highest probability. In the former case, this likely reflects the fact that robberies are usually perpetrated by strangers (15). In the latter case, filing insurance claims is usually dependent upon an official police report, creating extra incentive to call the police. Examining the effects of race on crime reporting, Xie and Lauritsen (16) found that assaults were more likely to be reported (by the victim) when the victim and offender were both Black, relative to White-on-White assaults. Assaults were less likely to be reported to the police when there was a Black victim and a White offender. Black-on-White assaults were reported at rates similar to the reference case. Witnesses may exercise a different set of principles when calling the police. Third parties not involved in the crime appear more likely to call when they know the suspect or victim (11), but less likely to call when the crime is less serious (17). Third parties appear to be less likely to call the police when they are Black (18).

Finally, the willingness of the public to cooperate with the police may be conditioned on the character of the actions taken by the police. Mazerolle et al. (19), for example, examined experimentally how formal procedural justice practices, implemented during brief traffic stops, impacted perceptions of police legitimacy. They found significant positive improvements in the treatment group in people's perceptions about their own encounter, but also about police legitimacy in general. The willingness to cooperate appears to be malleable in response to an individual's own experience with the police (20). Brantingham and Uchida (3) examined how calls-for-service changed following police activity surrounding homicide events. As Mazeroll et al. (19), the focus was on the public response to routine policing events occurring in their local community. Brantingham and Uchida (3) found that calls-for-service increase locally in the aftermath of homicides, which is at least consistent with the conclusion that trust in policing does not fall below some threshold in response to these acute and intensive police actions [see refs (21, 22)]. At a broader scale, Desmond et al. (1) examined the community response to instances of unjust police action that became widely known through media reporting. They focus the 2004 October beating of a Black man, Frank Jude, by Milwaukee police officers. They found that police calls-for-service fell significantly once news of the beating broke, five months later, but also that calls rebounded over the course of the year. A reanalysis of the data, however, found that the results were heavily influenced by an outlier and the functional form of the model estimated (23). Once removed, the data suggested that calls to police did not change significantly in response to the beating of Frank Jude. Moyer (24) examined whether the death of Freddie Grey in 2015 April, while in the custody of the Baltimore Police, and subsequent protests, led to change in community calls to the police for vehicle accidents, adult well-being checks, behavioral health, and other non-crime-related matters. Moyer found that calls to the police for non-crime-related matters in Baltimore, MD, did not change across the entire city or by the sections of the city that varied based on levels of poverty, age, race, employment, poverty, and housing vacancy following the death of Freddie Grey.

## The murder of George Floyd and the social response

The murder of George on 2020 May 25 by a Minneapolis police officer is by now a familiar story to all. The familiarity is in part the result of a bystander video that captured the entire event. The viral spread of the video, over both new and traditional media, caused a surge in online activism and ignited street-based protests across the United States and many locations around the world. #Blacklivesmatter was tweeted more than 8.8 million times on 2020 May 28 alone, three days after Floyd's death (25). Surveys suggest that 15–26 million Americans engaged in street protests in the subsequent weeks (5). Protests on- and offline captured widespread moral outrage (26) and focused attention on other instances of police violence. Emerging out of the social response were calls for immediate changes to the practice of policing ranging from incremental reforms to the #defundthepolice movement (6, 27).

The full impact of George Floyd's murder on policing in America will not be known for quite some time. Nevertheless, it is fair to say that public opinion about policing was at or near its lowest point in decades shortly following the murder and that this sentiment was widespread. Reny and Newman (28), for example, found in opinion surveys that the murder of George Floyd was associated with sharp jump in unfavorable opinions of police among politically liberal Whites, as well as Black, Hispanic, and Asian Americans. While the prior literature shows little impact of highly publicized police abuse or deadly force cases on the public's willingness to call the police (1, 23, 24), there are few incidents that have had such widespread impact on public perceptions of the police as the murder of George Floyd. If an effect did materialize, it is reasonable to expect that the impact was immediate, tracking public opinion. This is the empirical issue we now address through an examination of how police calls-for-service in Los Angeles and New York changed in response to the murder of George Floyd.

## Data and methods

### Data

The analyses presented below focus on police calls-for-service from the City of Los Angeles and the City of New York between 2019 and late 2021. Call data were extracted from the open data portals maintained by each city (https://data.lacity.org,https://data.cityofnewyork.us). We extracted calls that originated from members of the public (i.e. not initiated by police) and corresponding to violent crime, property crime, and quality-of-life disorder. Violent crime calls include robbery, battery (i.e. simple assault), aggravated assault (i.e. assault with a deadly weapon), and “shots fired.” Property crime calls include burglary, personal theft, and car theft. In Los Angeles, quality-of-life calls include intoxication, major disturbance, minor disturbance, vandalism, dispute, and screaming. In New York, quality-of-life calls include observing narcotics use and sale, criminal mischief, graffiti and vandalism, disorderly conduct, and dispute. The calls were aggregated by week and census tract. In Los Angeles, the mapping to census tract was based on the latitude and longitude of centroid of the Los Angeles Police Department (LAPD) reporting district (RD) in which each call occurred (see^3, 29^). In New York, the mapping was based on the latitude and longitude of the midpoint of the street segment on which the crime occurred. Demographics characteristics of census tract were obtained from the American Community Survey. We define census tracts as Asian, Black, Hispanic, or White, based on whether more than 50% of the residents living in that tract report being one of these race or ethnic groups. Because Los Angeles and New York are diverse, multi-ethnic cities, we also recognize non-majority census tracts as those where no one racial-ethnic group exceeds 50% of the population. Thus, our analyses cover all but two census tracts in Los Angeles and all but one census tract in New York. We also present results for all census tracts combined, which shows that the overall effects are substantively similar to the effects broken down by race–ethnicity. Research has noted considerable errors in police calls-for-service data (30, 31). Occasionally, the event identified by call type does not always represent what happened on the ground. Since our question concerns the willingness of members of the public to cooperate with the police in the co-production of public safety, the veracity of the call with respect to actual events is of less concern.

We collected police calls-for-service in Los Angeles spanning the period between 2019 January 1 and 2021 November 21, representing a total of 150 consecutive weeks of observation. During this period, the LAPD fielded 407,817 violent crime calls-for-service, 201,527 property crime calls-for service, and 1,001,654 quality-of-life calls-for-service. The combined 1,610,998 calls represent a call volume of about 10,740 calls-for-service per week related to these call types alone. We collected police calls-for-service for New York City spanning the period between 2019 January 1 and 2021 October 10, representing 144 consecutive weeks of observation. Over this period, the New York Police Department (NYPD) fielded 450,165 violent crime calls-for-service, 194,937 property crime calls-for-service, and 1,696,970 quality-of-life calls-for-service. The combined 2,342,092 calls represent a call volume of 16,255 calls per week for these call types alone.

### Measures

We use a regression discontinuity design (RDD) to examine the impact of the murder of George Floyd on police calls-for-service. RDD models assume that observational units on either side of a treatment cutoff are sufficiently similar in unobserved characteristics that they can be compared as if their treatment status was randomly assigned at that discontinuity (32–36). The treatment discontinuity may be the implementation of some new policy at a time *c* and observations of units in some small-time window just before (*c–t*) serve as control for those same units observed in some small-time window just after (*c *+ *t*) the onset of treatment. The discontinuity might be spatial (e.g. 35) or correspond to some eligibility criterion that separates units into treated and untreated groups. In the present case, the treatment discontinuity is the murder of George Floyd on 2020 May 25. We assume that the baseline environmental and socio-political neighborhood conditions that drive police calls-for-service were largely identical in the weeks immediately prior to, and immediately after George Floyd's murder. This assumption is particularly important, given, the putative impact that the global COVID-19 pandemic on crime and disorder. The impact appears to have varied by crime type and between cities (37, 38). RDD is advantageous in this situation precisely because the impact of the pandemic on crime and disorder, as well as police calls-for-service, were present in the weeks just before and after the murder of George Floyd (the treatment discontinuity). Any changes observed in police calls-for-service therefore represent the unique effect that the George Floyd murder and subsequent changes in trust in the police had on the process that generates calls to the police. We estimate the changes in calls for service using a range of alternative parameterizations of the RDD local linear regression model (36). We also compare how sensitive our estimates are to the choice of the week in which Floyd was murdered using placebo tests that randomly assign the discontinuity to different weeks. Given the inherent volatility in police calls-for-service, it is possible that random assignment of discontinuities would produce similar effect size estimates without any true causal effect (see 39, 40). We count the number of random assignments (out of 1,000) with an effect at least as large that observed for the week of George Floyd's murder as a guide for the false-positive rate. We rely on Bonferroni corrected *P*-values to guard against false-discoveries.

## Results

We report the mean number calls per week per census tract where one race–ethnicity (Asian, Black, Hispanic, and White) makes up a majority (≥50%) of the population, and where no one race–ethnicity reaches this threshold (non-majority) (see Table S1 in the “Supplementary Material”). Shown are means for the 73 weeks before the murder of George Floyd and the 77 weeks after (71 weeks in New York). In Los Angeles, violent crime calls-for-service are nominally higher, while property crime and quality-of-life crime calls are nominally lower after the murder of Floyd. In New York, property crime calls-for-service are nominally higher, while changes in violent crime and quality-of-life crime calls appear mixed.

### Violent crime calls-for-service

**Fig. 1.**
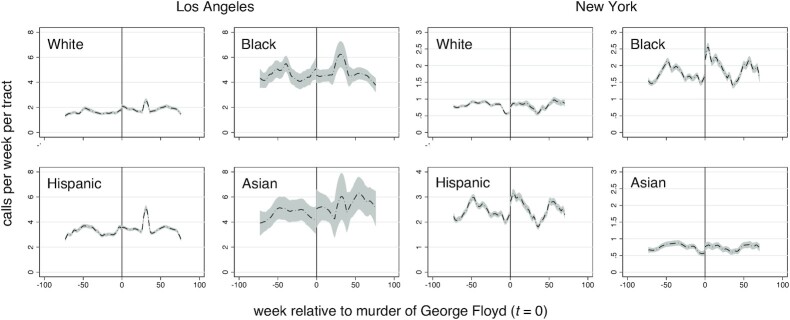
Violent crime calls-for-service per census tract by majority population in Los Angeles and New York. Shown is the local polynomial smoothed line with 95% confidence intervals (CI)..

**Fig. 2.**
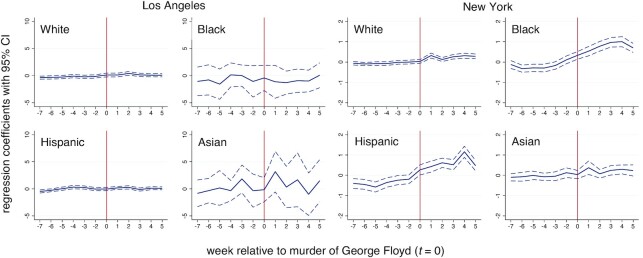
Period specific regression coefficients along with 95% CIs for violent crime calls-for-service in Los Angeles and New York census tracts by race–ethnicity.

Figure 1 shows the local polynomial smoothed violent crime calls-for-service in Los Angeles and New York census tracts by majority population group. Numerical results are presented for non-majority census tracts. The cutoff at *t *= 0 corresponds to the murder of George Floyd. Calls-for-service are volatile over time in both cities, but display more weekly regularity in New York compared with Los Angeles. Visual inspection suggests that decreases in violent crime calls-for-service occurred at the discontinuity in Black majority census tracts and increases in Asian majority census tracts in Los Angeles. Other shifts in Los Angeles and New York are difficult to distinguish from normal variation. Table 1 present RDD model estimates for the average treatment effects at the cutoff. Small increases in violent crime calls in White and Asian majority as well as non-majority tracts, and small decreases in violent crime calls in Black and Hispanic majority tracts in Los Angeles are all not significant, according to estimates using robust standard errors (SEs; with or without Bonferroni correction). By contrast, small increases in violent crime calls in White and Black majority census tracts in New York are significant. These amount to approximately 0.2 and 0.5 additional violent crime calls-for-service per week per tract, respectively (Table 1). Combining all census tracts suggests that the murder of George Floyd did not impact overall violent crime calls-for-service in Los Angeles or New York. Figure 2 shows the period specific regression coefficients (29). The temporal trends suggest that the increases were relatively short-lived. One-sided placebo tests are consistent with measures of significance based on conventional SEs (Table 1).

**Table 1.** RDD models estimates of the average treatment effect at the cutoff for violent crime calls-for-service.

	Los Angeles						New York					
	White	Black	Hispanic	Asian	Non-majority	Overall	White	Black	Hispanic	Asian	Noin-majority	Overall
Conventional	0.266*	−0.638	−0.121	1.332	0.267	0.0696	0.196***	0.533***	0.236	0.256**	0.198*	0.158*
	[0.0275, 0.505]	[−2.139, 0.863]	[−0.384, 0.143]	[−0.836, 3.500]	[−0.0875, 0.622]	[−0.120, 0.260]	[0.110, 0.282]	[0.315, 0.750]	[−0.0888, 0.561]	[0.0931, 0.420]	[0.0124, 0.384]	[0.00569, 0.310]
Bias-corrected	0.225	−0.713	−0.156	1.568	0.237	0.0421	0.177***	0.485***	0.142	0.246**	0.151	0.117
	[−0.0143, 0.463]	[−2.214, 0.788]	[−0.420, 0.107]	[−0.600, 3.736]	[−0.118, 0.592]	[−0.148, 0.232]	[0.0910, 0.263]	[0.267, 0.702]	[−0.182, 0.467]	[0.0831, 0.410]	[−0.0351, 0.337]	[−0.0353, 0.269]
Robust	0.225	−0.713	−0.156	1.568	0.237	0.0421	0.177***	0.485***	0.142	0.246*	0.151	0.117
	[−0.0565, 0.505]	[−2.547, 1.121]	[−0.473, 0.160]	[−1.005, 4.141]	[−0.191, 0.665]	[−0.187, 0.271]	[0.0801, 0.273]	[0.242, 0.728]	[−0.209, 0.493]	[0.0505, 0.442]	[−0.0501, 0.352]	[−0.0423, 0.276]
*N* tract weeks	35,850	2,100	64,500	900	36,600	140,400	95,876	60,992	58,280	15,840	74,856	310,676
One-sided placebo test *P*^†^	0.026	0.222	0.889	0.82	0.1	–	0.001	<0.001	0.08	0.005	0.018	–

95% CIs in brackets; *P < 0.05, **P< 0.01, and ***P < 0.001; Bonferroni correction for 24 hypotheses assuming *ɑ* < 0.05 requires *P* < 0.0021. ^†^Empirical *P*-value from data permutations testing the hypothesis that a shift in calls (negative or positive) at least as large could be observed for 10^3^ randomly chosen cutoff points across the data timeline.

### Property crime calls-for-service

**Fig. 3.**
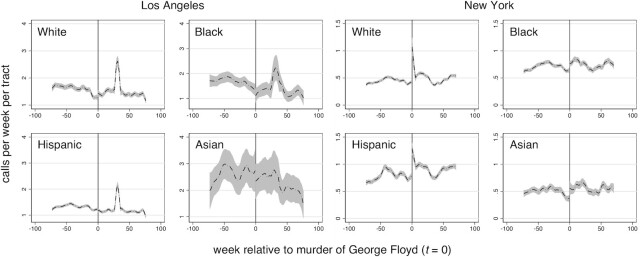
Property crime calls-for-service per census tract by majority population in Los Angeles and New York. Shown is the local polynomial smoothed line with 95% CI.

**Fig. 4.**
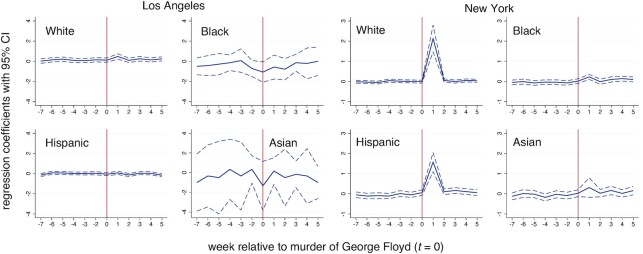
Period specific regression coefficients along with 95% CIs for property crime calls-for-service in Los Angeles and New York census tracts by race–ethnicity.

Figure 3 shows the polynomial smoothed property crime calls-for-service in Los Angeles and New York census tracts by majority population group. Property crime calls do not show an obvious departure at the cutoff in Los Angeles. A later peak in the time series corresponds to heavy calls-for-service associated with New Year celebrations. By contrast, White and Hispanic census tracts in New York show a large increase in property crime calls-for-service immediately following the murder of George Floyd. Increases in Black and Asian tracts are less obviously different from the seasonal pattern. Table 2 confirm these visual interpretations. The small increase in property crime calls-for-service in White majority and non-majority census tracts, and small decreases in property crime calls in Black, Hispanic, and Asian tracts in Los Angeles are all non-significant (with or without Bonferroni corrections). White, Hispanic, and non-majority tracts in New York exhibited a significant increase in property crime calls-for-service. These amounted to an increase of 0.7, 0.6, and 0.1 calls-for-service per week per tract, respectively (Table 2). Black and Asian tracts in New York experienced an increase that is significant without Bonferroni correction, but not with correction. Here, we observed increases of 0.1 and 0.2 calls per week per tract, respectively. Combining all census tracts shows a significant increase of 0.4 calls per week per tract only in New York. Figure 4 illustrates that the temporal trends do not deviate in Los Angles over the medium-term. In New York, the increases in property crime calls-for-service are also short-lived. One-sided placebo tests are consistent with measures of significance based on conventional SEs (Table 2).

**Table 2.** RDD models estimates of the average treatment effect at the cutoff for property crime calls-for-service.

	Los Angeles						New York					
	White	Black	Hispanic	Asian	Non-majority	Overall	White	Black	Hispanic	Asian	Non-majority	Overall
Conventional	0.168	−0.28	−0.0458	−0.36	0.171	0.0648	0.686***	0.120**	0.533***	0.197**	0.127***	0.352***
	[−0.0112, 0.347]	[−0.772, 0.213]	[−0.179, 0.0875]	[−1.625, 0.906]	[−0.00491, 0.347]	[−0.0450, 0.175]	[0.449, 0.922]	[0.0366, 0.202]	[0.371, 0.696]	[0.0638, 0.329]	[0.0554, 0.199]	[0.272, 0.431]
Bias-corrected	0.163	−0.346	−0.0599	−0.32	0.16	0.0601	0.715***	0.104*	0.552***	0.194**	0.114**	0.357***
	[−0.0159, 0.343]	[−0.839, 0.146]	[−0.193, 0.0734]	[−1.586, 0.946]	[−0.0158, 0.336]	[−0.0497, 0.170]	[0.478, 0.951]	[0.0215, 0.187]	[0.389, 0.714]	[0.0614, 0.327]	[0.0428, 0.186]	[0.278, 0.437]
Robust	0.163	−0.346	−0.0599	−0.32	0.16	0.0601	0.715***	0.104*	0.552***	0.194*	0.114**	0.357***
	[−0.0535, 0.380]	[−0.933, 0.240]	[−0.222, 0.103]	[−1.854, 1.214]	[−0.0523, 0.373]	[−0.0750, 0.195]	[0.462, 0.967]	[0.0101, 0.199]	[0.369, 0.735]	[0.0339, 0.354]	[0.0325, 0.196]	[0.268, 0.446]
*N* tract weeks	35,850	2,100	64,500	900	36,600	140,250	95,628	60,532	58,280	15,840	74,724	309,404
One-sided placebo test *P*^†^	0.051	0.176	0.163	0.328	0.065	–	<0.001	0.016	<0.001	0.004	0.002	–

95% CIs in brackets; *P < 0.05, **P< 0.01, and ***P< 0.001; Bonferroni correction for 24 hypotheses assuming *ɑ* < 0.05 requires *P* < 0.0021. ^†^Empirical *P*-value from data permutations testing the hypothesis that a shift in calls (negative or positive) at least as large could be observed for 10^3^ randomly chosen cutoff points across the data timeline.

### Quality-of-life crime calls-for-service

**Fig. 5.**
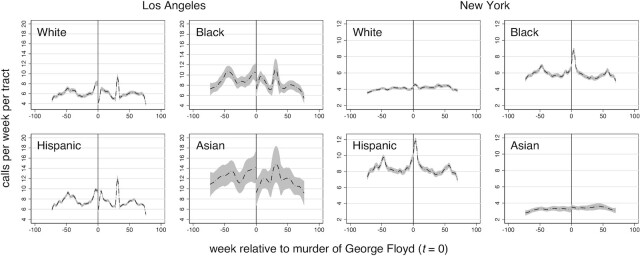
Quality-of-life crime calls-for-service per census tract by majority population in Los Angeles and New York. Shown is the local polynomial smoothed line with 95% CI.

**Fig. 6.**
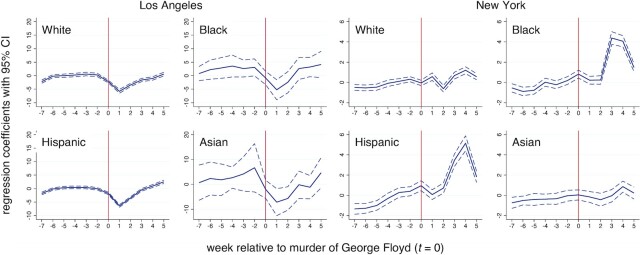
Period specific regression coefficients along with 95% CIs for quality-of-life crime calls-for-service in Los Angeles and New York census tracts by race–ethnicity.

Figure 5 shows the polynomial smoothed quality-of-life crime calls-for-service. Visual inspection suggests that public calls to the police moved in opposite directions in Los Angeles and New York. In Los Angeles, White, Black, Hispanic, and Asian majority census tracts showed steep drops in quality-of-life calls. In New York, sharp increases are seen in Black and Hispanic census tracts, with increases in White and Asian tracts less obviously different from general seasonal variability. These observations are confirmed in RDD estimates of the average treatment effects. Table 3 documents significant decreases in calls in White, Hispanic, and non-majority tracts in Los Angeles, and significant increases in Black, Hispanic, and non-majority tracts in New York. The drops in Los Angeles were equivalent to around 4.5, 4.2, and 3.7 fewer calls for White, Hispanic, and non-majority tracts, respectively (Table 3). In New York, the increases were equivalent to about 1.7, 1.8, and 1.0 more calls in Black, Hispanic, and non-majority tracts, respectively. Combining all census tracts shows a significant reduction of 3.7 quality-of-life calls per week per tract in Los Angeles and a significant increase of 0.9 calls per week per tract in New York. Figure 6 shows the temporal trends before and after the murder of George Floyd. The declines in quality-of-life calls-for-service rebound in two to five weeks, depending upon the community, lagging the longest in White and Hispanic census tracts and rebounding the fastest in Black and Asian tracts. In New York, the temporal trends are more complex, oscillating, and then peaking four to five weeks after the murder. One-sided placebo tests are consistent with measures of significance based on conventional SEs (Table 3).

**Table 3.** RDD models estimates of the average treatment effect at the cutoff for quality-of-life offense calls-for-service.

	Los Angeles						New York					
	White	Black	Hispanic	Asian	Non-majority	Overall	White	Black	Hispanic	Asian	Non-majority	Overall
Conventional	−4.291***	−3.213**	−4.031***	−5.581*	−3.561***	−3.648***	0.374**	1.744***	1.816***	0.196	1.022***	0.960***
	[−5.203, −3.379]	[−5.600, −0.826]	[−4.673, −3.389]	[−10.28, −0.877]	[−4.352, −2.771]	[−4.214, −3.083]	[0.0901, 0.659]	[1.290, 2.198]	[1.279, 2.353]	[−0.336, 0.728]	[0.624, 1.420]	[0.714, 1.205]
Bias-corrected	−4.488***	−3.620**	−4.147***	−5.866*	−3.733***	−3.685***	0.372*	1.714***	1.765***	0.231	0.995***	0.898***
	[−5.400, −3.577]	[−6.007, −1.233]	[−4.789, −3.505]	[−10.57, −1.162]	[−4.524, −2.943]	[−4.250, −3.120]	[0.0883, 0.657]	[1.260, 2.168]	[1.228, 2.303]	[−0.301, 0.763]	[0.596, 1.393]	[0.653, 1.144]
Robust	−4.488***	−3.620*	−4.147***	−5.866*	−3.733***	−3.685***	0.372*	1.714***	1.765***	0.231	0.995***	0.898***
	[−5.464, −3.513]	[−6.394, −0.847]	[−4.818, −3.476]	[−11.51, −0.225]	[−4.576, −2.890]	[−4.288, −3.082]	[0.0281, 0.717]	[1.168, 2.260]	[1.117, 2.414]	[−0.407, 0.869]	[0.514, 1.476]	[0.617, 1.180]
*N* tract weeks	36,000	2,100	64,650	900	36,750	140,700	96,192	60,912	58,464	15,840	75,456	306,864
One-sided placebo test *P*^†^	<0.001	0.003	<0.001	0.036	<0.001	–	0.028	<0.001	<0.001	0.281	<0.001	–

95% CIs in brackets; *P < 0.05, **P < 0.01, and***P < 0.001; Bonferroni correction for 24 hypotheses assuming *ɑ* < 0.05 requires *P* < 0.0021. ^†^Empirical *P*-value from data permutations testing the hypothesis that a shift in calls (negative or positive) at least as large could be observed for 10^3^ randomly chosen cutoff points across the data timeline.

## Discussion and conclusions

The murder of George Floyd triggered widespread protest in the United States and beyond. The movement led to intense scrutiny of police practices and resulted in high-profile calls to “defund the police.” While prior studies have shown that cooperation with police is not dependent upon trust (2, 8), nor is it particularly responsive to public opinion following other publicized cases of abusive police use of force (23, 24), the possibility that the murder of George Floyd and the subsequent fallout caused a “state change” needed evaluation. Perhaps, the murder of George Floyd was the final straw driving people to consider alternatives to policing in dealing with the problems they confront daily.

The analyses presented here suggest that the effects on police calls-for-service were limited to only certain call types and frequently were in the opposite direction of popular narratives on how a decline in trust in the police would lead the public to stop calling the police. We focused separately on violent crime, property crime, and quality-of-life calls-for-service because of the expectation that people have greater discretion to *not* call the police when they observe or experience quality-of-life offenses (17). Thus, we expected that quality-of-life offense calls would be more responsive to the murder of George Floyd than property crime or violent crime calls. These expectations are partially supported. Specifically, in Los Angeles, violent and property crime calls did not change significantly, while quality-of-life calls fell sharply in the aftermath of the murder, consistent with the hypothesis of greater caller discretion for these call types. Importantly, in Los Angeles, quality-of-life calls fell furthest in White majority tracts (−73%), followed by Hispanic (−53%) and non-majority tracts (−50%); the declines in Asian (−47%) and Black tracts (−40%) were non-significant. On the face of it, this may suggest that people in White majority, Hispanic majority, and diverse non-majority areas of Los Angeles have greater discretion to *not* call the police compared with people in Asian and Black areas of the city. In New York, the pattern is quite different. Here, violent, property, and quality-of-life calls either increased or did not change significantly. Violent crime calls increased the most in Black (+28%), followed by White majority tracts (+22%) in the immediate aftermath of the murder. Property crime calls increased the most in White (+160%), followed by Hispanic majority tracts (+70%). Quality-of-life calls increase the most in Black (+29%), followed by Hispanic (+21%) and non-majority tracts (+20%). The shifts in New York may signal an increase in cooperation with police following the murder of George Floyd, an observation that runs counter to expectations that calls should fall in response to negative public opinion and reduced trust in police. A cynical interpretation of the results in New York would be that the murder of George Floyd triggered a spike in perceived “minority threat” (41), which then resulted in increased calls to the police *by* White residents *about* their Black and Hispanic neighbors in White as well as Black and Hispanic majority tracts. To account for the observed reduction in calls-for-service in Los Angeles, we would have to posit that White (and perhaps Asian) residents were checking themselves before calling the police on their Black and Hispanic neighbors. Individual-level data on the characteristics of callers are not available to examine these conjectures. However, research on crime victimization more broadly indicates that the reasons why individuals call (or do not call) the police are highly variable and perceived minority threat or lack of trust in the police are two of many possible motives (9). Whatever the case, the shifts in calls-for-service were relatively short-lived. By and large, both the declines in Los Angeles and increases in New York in calls lasted for two to five weeks before returning to their prior trends.

Why did the murder of George Floyd and the subsequent social movement not have more dramatic and longer-lasting effects on calls-for-service? One possibility is that the social movement, though clearly street-oriented and widespread at the start, returned to “hashtag activism” after a time (42). Attention was siphoned away, and people returned to their existing routines. In a parallel domain, for example, Nguyen et al. (43) found that tweets with negative sentiment referencing Black people declined by around 32% in the immediate aftermath of the George Floyd's murder, but also that the decline lasted just a few weeks. A pragmatic interpretation of the results is that the immediate needs of crime victims quickly eclipse more abstract goals of police reform. For example, while people may have had more negative opinions of the police following the murder of George Floyd (28, 44), they nevertheless had no real alternative but to call the police for assistance if they were the victim of an assault, had their car stolen, or were bothered by a loud party. Such pragmatic concerns are consistent with a 2020 Gallup Panel survey, where despite only 18% of Black Americans feeling “very confident” that they would receive courtesy and respect in a police encounter, 80% of Black Americans preferred that police spend the same or more time in their area (45). The murder of George Floyd and the subsequent protests did not drive up the perceived costs of interacting with the police higher than the perceived costs of not reporting violent, property, and quality-of-life offenses. This conclusion holds across communities.

One implication of this work is that the public at large may have limited patience or tolerance for experimentation with alternatives to policing, if that experimentation also means that no one is there to respond to the crime and disorder encountered today. The volume of calls that police handle is staggering, and many call types are not easily transferrable to alternative service providers (46). Public patience may be most tested in the heavily impacted minority neighborhoods, which must rely on the police to respond to crime. Racially segregated neighborhoods with greater concentrations of poverty also suffer from higher rates of crime. This may in part explain why minority communities in Minneapolis, MN, voted down a proposal to replace the Minneapolis Police Department with an alternative Department of Public Safety that was not clearly defined (47). This does not mean, however, that those same communities do not desire police reform. The downsides of an “incident-driven” policing model have long been recognized (48–50). Yet, rapid police response anywhere at any time is what the public has come to expect from the “incident-driven” model. Police reform models need to account for these expectations.

There are several limitations to this study. Calling the police is not a perfect proxy for trust in police, as recognized previously (2). Consequently, that calls to the police did not collapse following the murder of George Floyd does not necessarily mean that trust in police remained buoyant. All we can conclude is that cooperation with the police continued much as it did before. More light might be shed on the issue if direct measures of trust in police were collected alongside evidence of when people call (or do not call) the police. As a measure of cooperation with police, calls-for-service also suffer several limitations. There are many ways that the public might cooperate with the police that are not registered in any official call (51). People may provide help in investigations, information at crime scenes or in friendly interactions that emerge naturally out of normal social interactions, or in various formal settings designed to support the co-production of public safety. Future research will need to assess the degree to which calls-for-service provides a useful proxy for this broader arena of public–police cooperation.

There are also limitations inherent to causal inference using RDD. RDD observational studies rely on several assumptions including (1) that there was an abrupt, unambiguous environmental disruption or policy shift that constitutes a treatment intervention; (2) that the so-called running variable that determines treatment cannot be manipulated; (3) that outcome probabilities are continuous at the cutoff in the absence of treatment; and (4) that exposure groups are exchangeable near the boundary (32–34). All four assumptions appear to be met in the current case. The murder of George Floyd marked an abrupt shift in public opinion about police, the timing of the event was beyond any form of manipulation, and the baseline rate of calls to the police was continuous and exchangeable near the cutoff, as demonstrated here by placebo tests. Thus, RDD offers a sound basis for inferring the causal effect of the murder of George Floyd on calls-for-service. However, our results may be limited in their generalizability for several reasons that extend beyond the validity of assumptions of RDD. In particular, we recognize that the conditions of global COVID-19 pandemic may have played a role in amplifying the scale and scope of protests following the murder of George Floyd. With many people working from home and perpetually connected to the Internet, the potential for this egregious example of police violence to go viral online and spark street protests may have been much higher than in other previous cases of police brutality. It seems plausible then that the effects that we do observe in Los Angeles and New York are potentially unique to this one event conditioned on the background conditions created by the pandemic. In the absence of these unique pandemic conditions, the impact on calls-for-service might have been more muted, which would be consistent with the results of prior studies (23, 24). This suggests that it would be challenging to wring estimates of causal effects out of formal comparisons with earlier examples of police violence. An alternative approach might be to compare calls-for-service in cities that experienced social protests related to George Floyd's murder with those that did not. However, given the breadth of protests spanning both street-based and online settings, it might be difficult to identify control and treatment units that are clearly separable. Disentangling the variation in treatment conditions from variation in other potential confounds such as local COVID-19 policies would be challenging if not impossible. In our case, we circumvent such confounds by having the cities serve as their own controls. Thus, our results are informative not because we believe them to identify a consistent *average treatment effect* stemming from police violence—a typical goal in observational approaches to causal inference—but rather because they point to the substantial stability of calls-for-service even under extreme (and perhaps unique) disruptions that might otherwise be expected to produce a fundamental shift in public cooperation with the police. We conclude that, despite the widespread social response to the murder of George Floyd, trust in the police apparently did not fall below some putative threshold that would produce sharp and enduring reductions in the willingness of the public to call the police to report violent, property, and quality-of-life offenses.

## Supplementary Material

pgac189_Supplemental_File

## Data Availability

The data used in this study are available from the Los Angeles and New York City open data portals (https://data.lacity.org/ and https://data.cityofnewyork.us).

## Notes

**Competing Interests:** P.J.B. and G.M. serve on the board of Geolitica. J.M. serves on the federal monitoring committee on the settlement agreements in *Floyd et al*.,*v. City of New York*, et al., 08 Civ. 1034 (AT), *Ligon, et al*.,*v. City of New York*, et al., 12-CV-2274 (AT), and *Davis et al. v. City of New York*, et al., 10-CV00699 (AT). The analyses and opinions expressed in the article reflect those of the authors only and not any other entity.
