# Supplementary material for: Changes in public–police cooperation following the murder of George Floyd
Source: PNAS Nexus. 2022 Sep 10;1(5):pgac189. doi: 10.1093/pnasnexus/pgac189 (PMC9802335; doi:10.1093/pnasnexus/pgac189)
Supplement: pgac189_Supplemental_File [file pgac189_supplemental_file.pdf]

Supplementary Information

Changes in Public-Police Cooperation Following the Murder of George Floyd

P. Jeffrey Brantingham*, Department of Anthropology, 341 Haines Hall, UCLA, Los Angeles, CA 90230, USA, branting@ucla.edu

George Mohler, Computer Science Department
245 Beacon Street, Boston College, Chestnut Hill, Mass. 02467-3859, USA, mohlerg@bc.edu

John MacDonald, Department of Criminology, University of Pennsylvania, 558 McNeil Building, Philadelphia, PA 19104-6286, johnmm@sas.upenn.edu

*Corresponding Author

This PDF File Includes:

Table S1. Calls per week per census tract, by majority race-ethnicity of the tract, before and after the murder of George Floyd.

Table S1. Calls per week per census tract, by majority race-ethnicity of the tract, before and after the murder of George Floyd.

	Weeks 1 to 73			Weeks 74 to 150 (144, NY)		
**Los Angeles**	N tract weeks	Mean	Std. dev.	N tract weeks	Mean	Std. dev.
**Violent Crime Calls for Service**						
White	17,447	1.6	2.2	18,403	1.9	2.7
Black	1,022	4.6	3.6	1,078	4.8	5.2
Hispanic	31,390	3.3	3.1	33,110	3.5	3.9
Asian	438	4.7	4.2	462	5.4	5.9
Non-majority	17,812	2.9	4.0	18,788	3.2	4.4
**Property Crime Calls for Service**						
White	17,447	1.6	1.8	18,403	1.5	2.2
Black	1,022	1.7	1.5	1,078	1.4	1.9
Hispanic	31,390	1.3	1.5	33,110	1.2	1.7
Asian	438	2.6	2.4	462	2.3	2.3
Non-majority	17,812	1.7	2.1	18,788	1.5	2.1
**Quality-of-life Calls for Service**						
White	17,520	6.1	6.0	18,480	6.0	6.2
Black	1,022	9.1	6.0	1,078	8.1	6.5
Hispanic	31,463	7.8	5.2	33,187	7.4	6.1
Asian	438	12.5	9.5	462	11.6	10.6
Non-majority	17,885	7.4	6.8	18,865	6.9	7.0
**New York**						
**Violent Crime Calls for Service**						
White	48,701	0.8	1.4	47,175	0.8	2.0
Black	30,910	1.7	2.0	30,082	1.9	2.2
Hispanic	29,565	2.4	2.5	28,715	2.5	2.6
Asian	8,030	0.8	1.2	7,810	0.7	1.2
Non-majority	37,887	1.3	1.8	36,969	1.4	1.9
**Property Crime Calls for Service**						
White	48,462	0.4	0.8	47,166	0.5	1.4
Black	30,712	0.7	1.0	29,820	0.7	1.1

Hispanic	29,565	0.8	1.1	28,715	0.9	1.3
Asian	8,030	0.5	0.8	7,810	0.6	0.9
Non-majority	37,866	0.6	0.9	36,858	0.6	1.0
**Quality-of-life Calls for Service**						
White	48,764	4.0	5.8	47,428	4.3	6.3
Black	30,879	6.0	5.5	30,033	5.9	5.6
Hispanic	29,638	8.4	7.4	28,826	8.4	7.3
Asian	8,030	3.2	4.9	7,810	3.4	5.6
Non-majority	38,252	5.1	6.0	37,204	5.3	6.4
